# European real world trans-catheter aortic valve implantation: systematic review and meta-analysis of European national registries

**DOI:** 10.1186/s13019-016-0552-6

**Published:** 2016-11-29

**Authors:** G. Krasopoulos, F. Falconieri, U. Benedetto, J. Newton, R. Sayeed, R. Kharbanda, A. Banning

**Affiliations:** Oxford Heart Centre, Oxford University Hospitals, Headley Way, Headington, Oxford, OX3 9DU UK

**Keywords:** Transcatheter aortic valve implantation, TAVI, TAVR, Aortic stenosis, Registry, Metanalysis

## Abstract

**Objective:**

Transcatheter aortic valve implantation (TAVI) has been adopted rapidly in Europe. TAVI national registries can augment understanding of technologies and represent real-world experience, providing further clinical insights. We undertook a meta-analysis of published European national TAVI registries to assess current results following TAVI in Europe.

**Methods:**

Electronic databases were searched. The review focused on the comparison of the following TAVI strategies: transfemoral (TF) and transapical (TA) SAPIEN and CoreValve implantation. Individual event rates for outcomes of interest were pooled using a mixed effect model.

**Results:**

Seven European national TAVI registries (UK, Swiss, Belgium, Italy, Spain, France, Germany) were identified, including a total of 9786 patients who received TF-SAPIEN (*n* = 2885), TA-SAPIEN (*n* = 2252) and CoreValve (*n* = 4649) implantation. Pooled incidence of 30-day mortality was 0.08% [95% Confidence Interval (CI): 0.05–0.11], 0.12% [95% CI: 0.07–0.19] and 0.06% [95% CI: 0.03–0.11] for TF-SAPIEN, TA-SAPIEN and CoreValve respectively (test for subgroup difference *P* = 0.18); there was high heterogeneity across European countries. Pooled incidence of stroke was comparable among the TAVI strategies (test for subgroup difference *P* = 0.79); the incidence of post-procedural moderate paravalvular leak ≥ 2 (*P* = 0.9) was similar across groups. CoreValve implantation was associated with an increased risk of pacemaker implantation (0.22 [95% CI: 0.19–0.26]; test for subgroup difference *P* < 0.0001). The lowest 30-day mortality was associated with TAVI performed in Spain (b coefficient −4.3; *P* = 0.03), in Italy (b coefficient −2.1; *P* < 0.0001), in UK (b coefficient −1.95; *P* = 0.01) and in France (b coefficient −2.8; *P* = 0.03). The German registry has the highest mortality for every TAVI strategy amongst all other European registries and especially for the TA-SAPIEN group.

**Conclusions:**

Transarterial TAVI approaches were associated with a low early mortality regardless of the type of device used. There was marked heterogeneity among European countries for early mortality.

## Background

Dr Alain Cribier reported the first transcatheter aortic valve implantation (TAVI) procedure in 2002 [1]. Following the randomized trials Placement of Aortic Transcatheter Valves (PARTNER) A and B [2, 3], TAVI is now considered the standard of care for symptomatic patients with aortic valve stenosis that are either high-risk or have been turned down for conventional aortic valve replacement [4]. However, even within these groups, the patients enrolled into the trials were highly selected, and therefore may not reflect real-world patients requiring treatment.

In Europe patients who undergo TAVI are recorded into national databases which capture the majority of the high risk or inoperable cases, due to co-morbidities and/or frailty patients. Their analysis can therefore provide further insight and evidence into the effectiveness of TAVI in the real-world clinical practice of symptomatic patients with aortic stenosis who are not candidates for conventional aortic valve replacement due to co-morbidities and/or frailty [4]. Furthermore, mixed national registries report country specific results that may be affected by variations in national health policy and local referral practice, device performance and definitions thus accounting for otherwise inexplicable differences in outcome and complications.

The aim of this study is to obtain an insight into the role of TAVI in the treatment of high-risk patients with aortic stenosis in Europe by conducting a meta-analysis of European national registries, focusing on the three most commonly used TAVI procedures : the transfemoral and transapical balloon-expandable Edwards SAPIEN transcatheter heart valve (Edwards Lifesciences, Irvine, CA, USA) and the self-expanding Medtronic CoreValve (Medtronic, Minneapolis, MN, USA).

## Material and methods

### Design

The present review was performed according to the Cochrane Collaboration and PRISMA statements [5].

### Eligibility criteria

Inclusion criteria for this meta-analysis were (1) All European national registries reporting outcomes of patients undergoing TAVI; (2) TAVI procedures should have been performed using transfemoral (TF SAPIEN) and/or transapical (TA SAPIEN) balloon-expandable Edwards SAPIEN transcatheter heart valve and/or self-expanding Medtronic CoreValve.

Care was taken to ensure that studies selected did not result in duplication of data. Non-English language, review articles, and editorials were excluded. Studies that did not separate results for TF SAPIEN, TA SAPIEN and CoreValve were also excluded.

### Search strategy

A literature search was done on the 1^st^ of September 2014 using MEDLINE, EMBASE, and Web of Science to identify relevant articles. Search terms used the controlled vocabularies of MEDLINE and EMBASE alone or in combination with text words including “transcatheter aortic valve implantation”, “TAVI”, “registry”, “Europe”. References from the selected studies were manually searched to identify any other potentially suitable publications.

Two reviewers independently screened all studies for inclusion. Disagreements were resolved by consensus. Agreement between reviewers regarding study inclusion was assessed using the Cohen k statistic.

### Meta-analysed endpoints

All included studies were interrogated for the following endpoints: 30-day and one year mortality, incidence of stroke, incidence of pacemaker implantation and presence of post deployment moderate paravalvular leak (≥2, according to authors definition).

### Statistical analysis

Mixed effects meta-analysis was performed pooling all single registry proportions using the DerSimonian-Laird estimate for all outcomes according to the TAVI strategy used. The *I* ^2^ statistic was used to assess the heterogeneity across the reported results. *I* ^2^ values of 25 to 49%, 50 to 74%, and 75% or greater were used to indicative low, moderate, and high levels of heterogeneity [6]. Cochrane Q statistic was used as test for subgroup differences (random effects model). The multivariate meta-regression (mixed model) used was used to adjust the effect of different TAVI strategies for the following risk factors: patients risk profile according to mean Logistic Euroscore [7], the reporting European country, the total number of centres involved and the sponsorship of the registry by a TAVI valve manufacturer. R^2^ was used to estimate the amount of heterogeneity accounted for in the multivariate model. Publication bias was assessed using Begg & Mazumdar test. Trim-and-fill method was used for estimating and adjusting for the number and outcomes of missing studies. A *p* < 0.05 was used as the level of significance and 95% confidence intervals (95% CI) have been reported where appropriate.

R version 3.1.0 (R Core Team (2014 http://www.R-project.org/.) and meta package (Guido Schwarzer (2014) http://CRAN.R-project.org/package=meta) were used for all statistical analyses.

## Results

### Studies selection

**Fig. 1:**
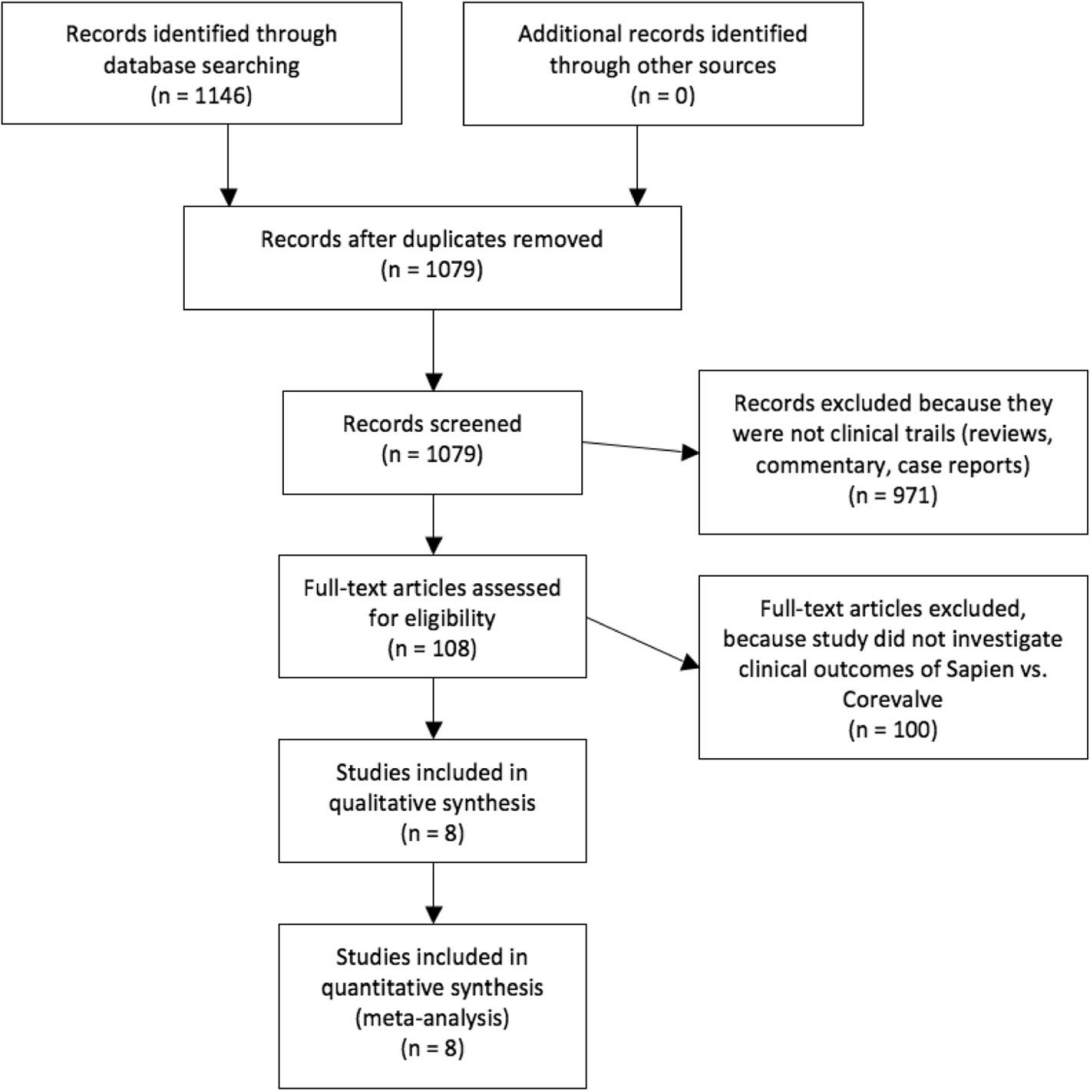
Outline of the systematic review process

From 1,079 abstracts, 108 full-text articles were assessed for eligibility. After evaluating the full-text articles, 8 met our eligibility criteria and were selected for the systematic review and meta-analysis [8–15]. The studies are summarized in Table 1 and depicted in Fig. 1. A Cohen k statistic of 90% was obtained for the final selection process.

**Table 1:** European registries overview

Registry	Period	TAVI technology	Number	Logistic EuroSCORE	Funded by manufacturer	N centers
Belgian National TAVI Registry [8]	2009–2010	SAPIEN TF	99	29	no stated	15
		SAPIEN TA	88	33		
		CoreValve	141	25		
FRANCE 2 TAVI Registry [9]	2010–2011	SAPIEN TF	1540	22.2	Yes	34
		SAPIEN TA	567	24.8		
		CoreValve	1043	21.3		
German TAVI Registry [10]	2009–2010	SAPIEN TF	123	20.3	Yes	22
		SAPIEN TA	113	20.3		
		CoreValve	1074	20.3		
Italian Registry TA TAVI [11]	2008–2012	SAPIEN TA	774	25.6	Yes	21
Italian Multicenter CoreValve Registry [12]	2007–2009	CoreValve	659	23	Yes	14
Spain TAVI Registry [13]	2010–2011	SAPIEN TF	504	17	no stated	44
		SAPIEN TA	302	19.2		
		CoreValve	610	16		
SWISS TAVI Registry [14]	2011–2013	SAPIEN TF	232	20.2	Yes	8
		CoreValve	324	20.2		
UK TAVI Registry [15]	2007–2010	SAPIEN TF	387	17.7	Yes	30
		SAPIEN TA	408	22.5		
		CoreValve	798	20.25		

*TAVI* transcatheter aortic valve implantation, *TF* transfemoral, *TA* transapical, *UK* United Kingdom

Seven European national TAVI registries (Belgium, France, Germany, Italy, Spain, Swiss, UK) were identified including 9786 patients which have received TF SAPIEN (*n* = 2885), TA SAPIEN (*n* = 2252) and CoreValve (*n* = 4649) implantation. All but Italian and Swiss registries reported on all three strategies. Two different and independent Italian TAVI registries reported on TA SAPIEN and CoreValve implantation separately. The Swiss registry reported on TF SAPIEN and CoreValve only. Mean logistic EuroSCORE ranged from 16% (Spain registry, CoreValve group) to 33% (Belgian registry, TA SAPIEN group).

### Meta-analysis

#### 30-day mortality

**Fig. 2:**
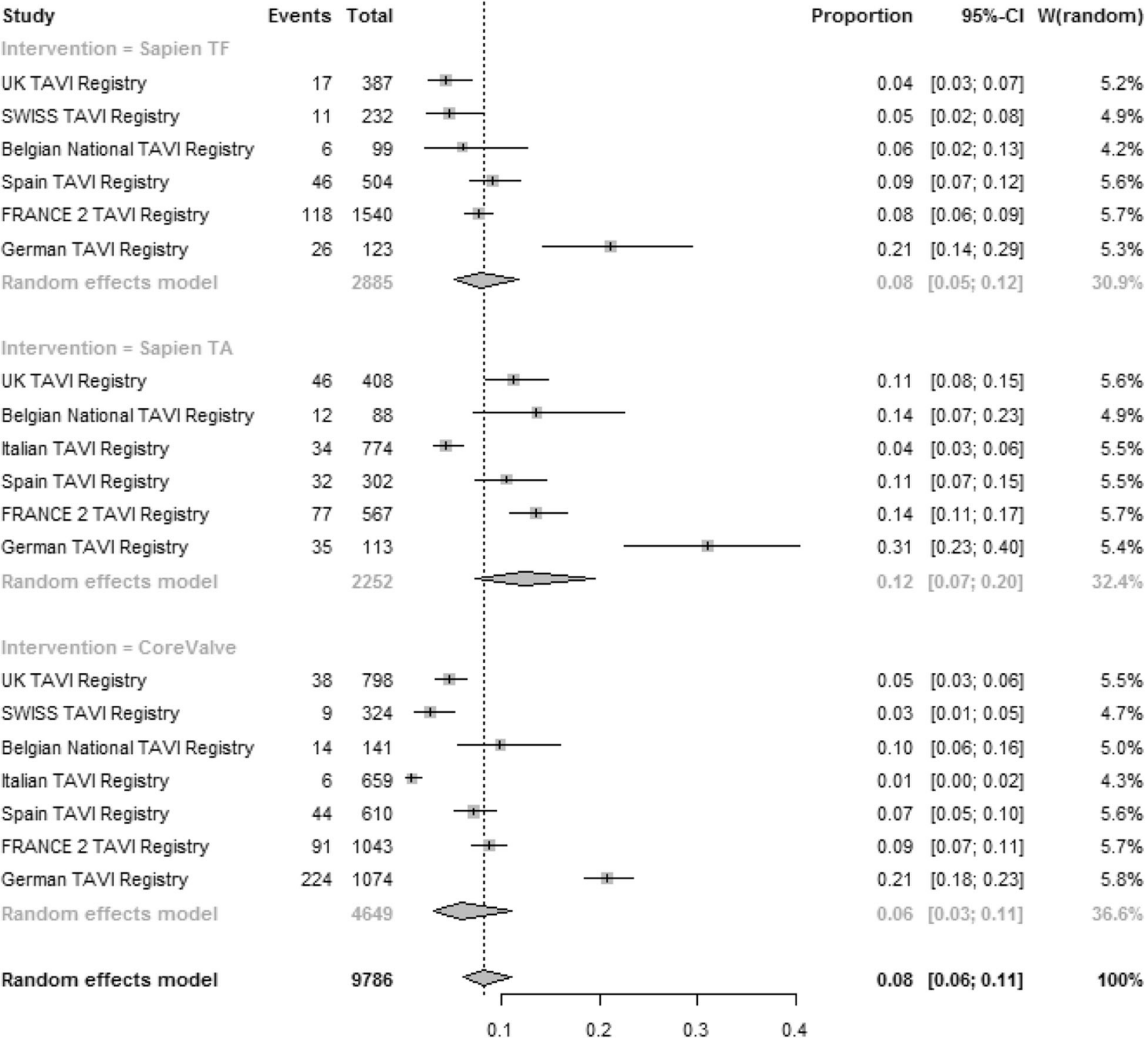
Meta-analysis for proportions of 30-day mortality

**Fig. 3:**
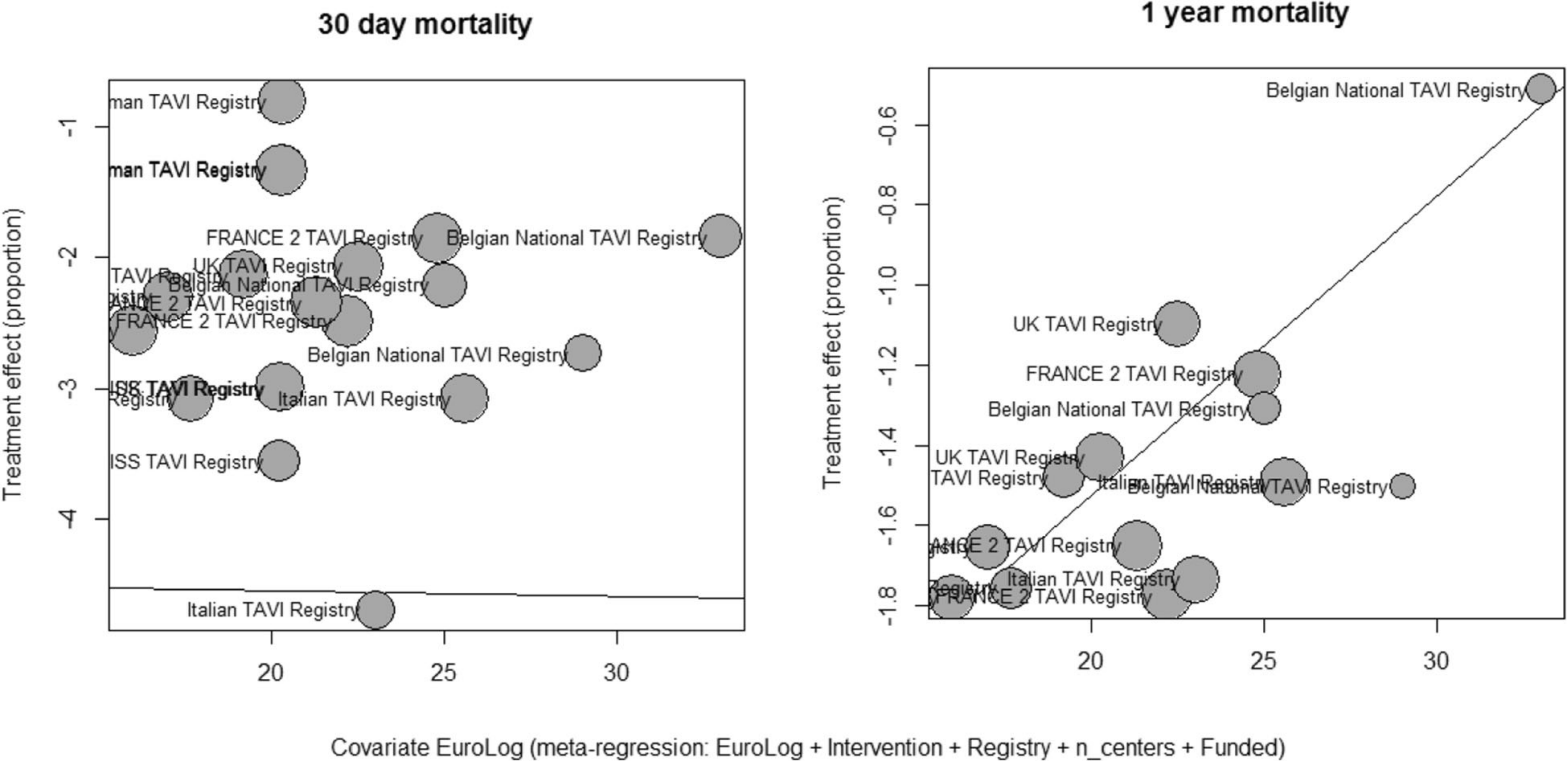
Bubble plot. Display the adjusted association between logistic EuroSCORE, 30-day mortality (left) and 1-year mortality (right)

The pooled estimate for 30-day mortality (Fig. 2) was 0.08 [95% CI: 0.05–0.11], 0.12 [95% CI: 0.07–0.19] and 0.06 [95% CI: 0.03–0.11] for TF SAPIEN™, TA SAPIEN and CoreValve respectively (test for subgroup difference *P* = 0.18). High heterogeneity was present among registries for all three strategies: TF SAPIEN (I^2^ = 86.6%), TA SAPIEN (I^2^ = 93.3%) and CoreValve (I^2^ = 97%). In multivariate meta-regression the increased risk for 30-day mortality was independently associated with TA SAPIEN strategy (b coefficient 0.60; *P* = 0.001). A higher 30-day mortality was reported from registries with higher number of centres involved into the national TAVI program (b coefficient 0.14; *P* = 0.03). The lowest 30-day mortality was associated with TAVI performed in Spain (b coefficient −4.3; *P* = 0.03), in Italy (b coefficient −2.1; *P* < 0.0001), in UK (b coefficient −1.95; *P* = 0.01) and in France (b coefficient −2.8; *P* = 0.03). The German registry has the highest mortality for every TAVI strategy amongst all other European registries and especially for the TA SAPIEN group. Patients risk profile assessed by the mean Logistic EuroSCORE was not associated with observed 30-day mortality (*P* = 0.9, Fig. 3, left). Moderators included in the multivariate model accounted for 98% amount of heterogeneity with no significant residual heterogeneity (*P* = 0.25). No publication bias was detected (*P* = 0.31).

### Stroke

**Fig. 4:**
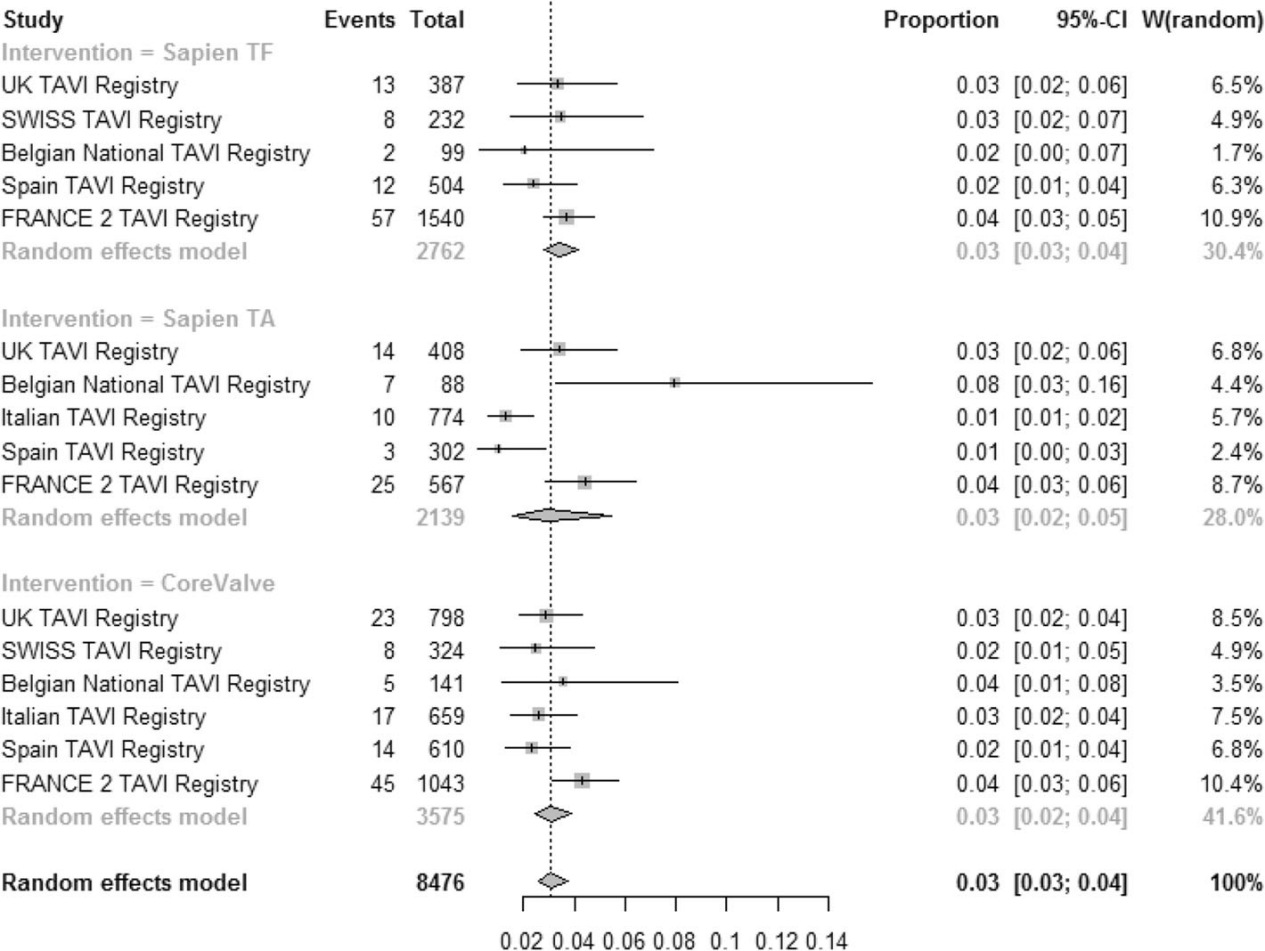
Meta-analysis for proportions of stroke

Pooled estimate for the incidence of stroke (Fig. 4) was 0.03 [95% CI: 0.03–0.04], 0.03 [95% CI: 0.02–0.05] and 0.03 [0.02–0.04] for TF SAPIEN™, TA SAPIEN and CoreValve respectively (test for subgroup difference *P* = 0.79). High heterogeneity was found among registries for TA SAPIEN (I^2^ = 81.2%) whilst TF SAPIEN and CoreValve were associate with low heterogeneity for stroke incidence (I^2^ = 0% and I^2^ = 32.6% respectively). The lowest incidence of stroke was associated with TAVIs performed in Italy (b coefficient −1.5; *P* = 0.02). Patients risk profile assessed by the mean Logistic EuroSCORE was not associated with the incidence of stroke (*P* = 0.74). Moderators included in the multivariate model accounted for 82.4% amount of heterogeneity with no significant residual heterogeneity (*P* = 0.32). No publication bias were detected (*P* = 0.27).

### Need for pacemaker implantation

**Fig. 5:**
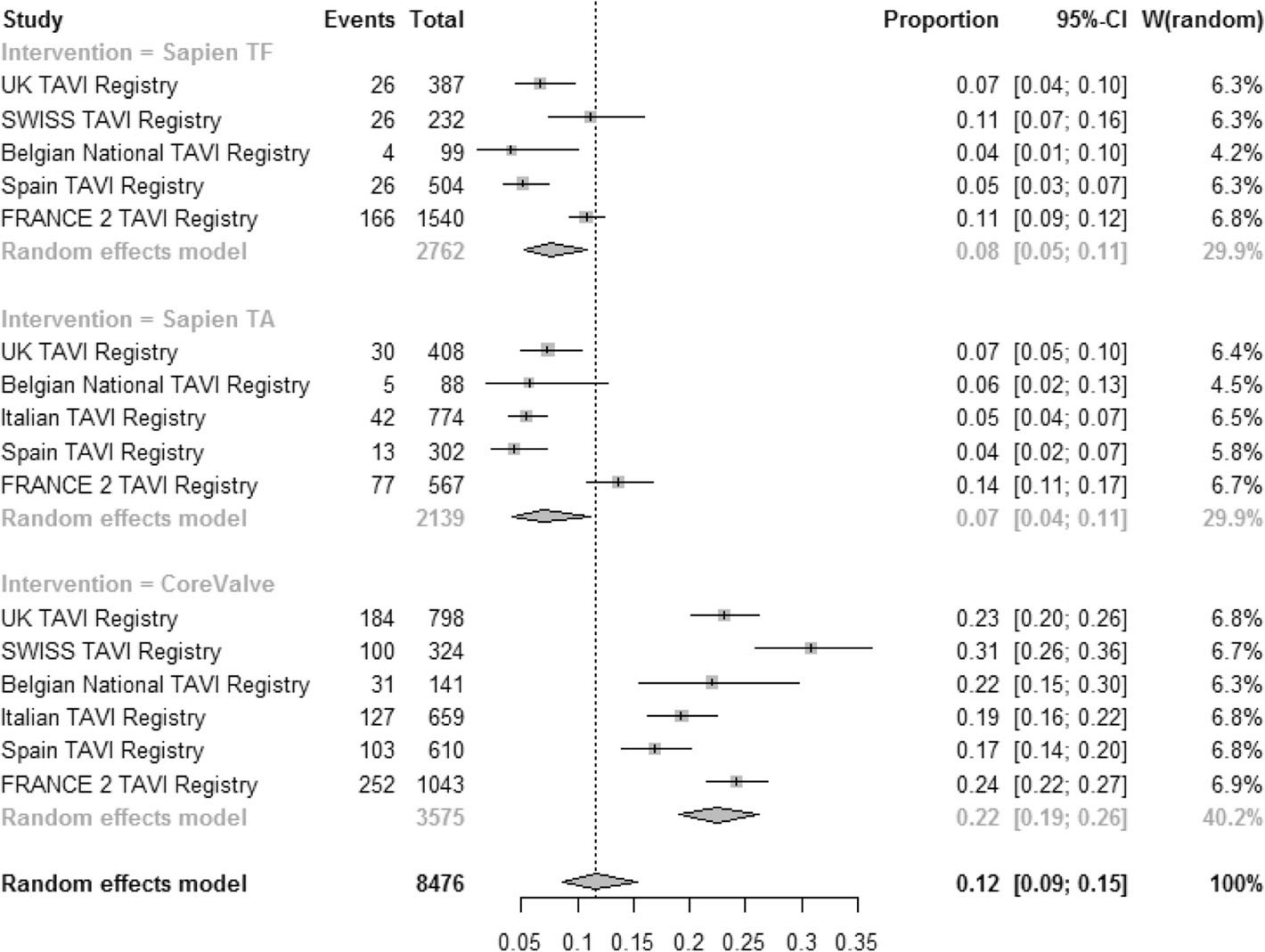
Meta-analysis for proportions of pacemaker requirement

Pooled estimate for incidence of pacemaker implantation (Fig. 5) was 0.08 [95% CI: 0.05–0.11], 0.07 [95% CI: 0.04–0.11] and 0.22 [95% CI: 0.19–0.26] for TF SAPIEN™, TA SAPIEN and CoreValve respectively (test for subgroup difference *P* < 0.0001). High heterogeneity was found among registries for TF SAPIEN (I^2^ = 80.9%), TA SAPIEN (I^2^ = 88.8%) and CoreValve (I^2^ = 83%) technologies. TF SAPIEN (b coefficient −1.2; *P* < 0.0001) and TA SAPIEN (b coefficient −1.08; *P* = 0.001) were independently associated with the lowest risk of pacemaker implantation. Patients risk profile assessed by the mean Logistic EuroSCORE was not associated with the rate of pacemaker implantation (*P* = 0.78). Moderators included in the multivariate model accounted for 90.5% amount of heterogeneity but significant residual heterogeneity was found (*P* = 0.02). No publication bias was detected (*P* = 0.08).

### Post implantation paravalvular leak

**Fig. 6:**
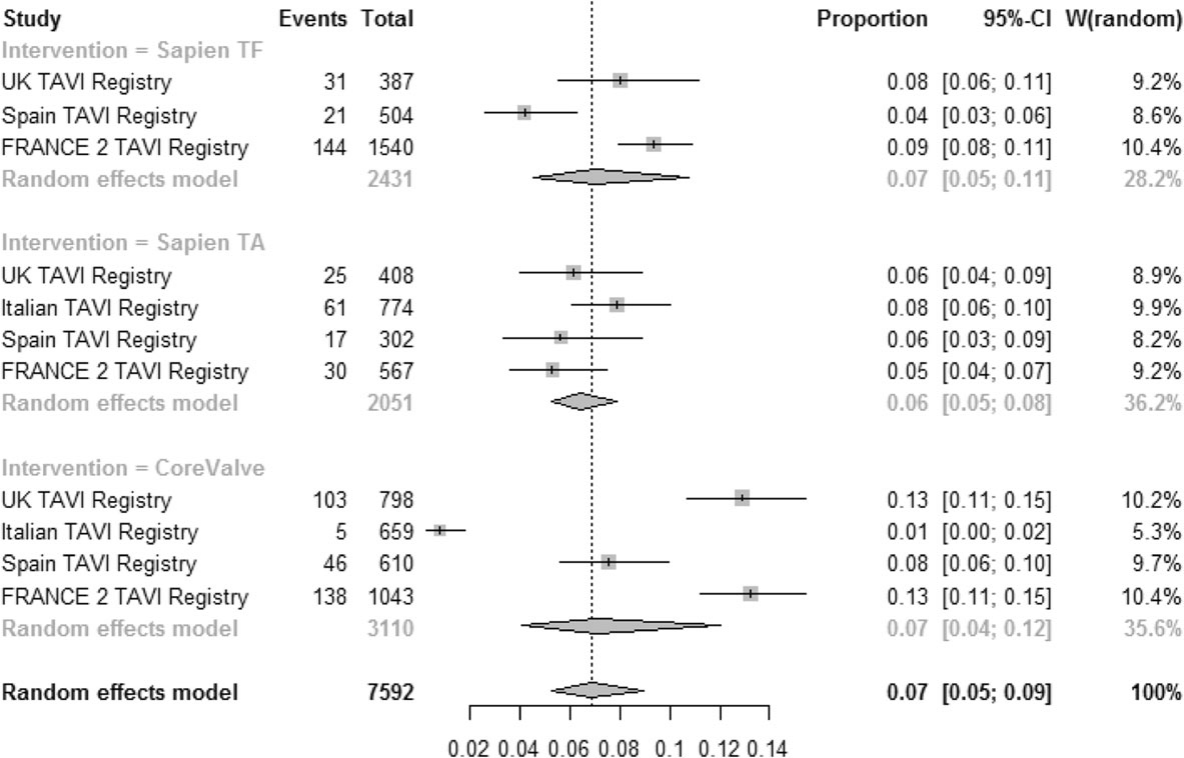
Meta-analysis for proportions of Incidence of paravalvular leak ≥ 2

There was no significant difference in the incidence of moderate paravalvular leak (≥2): 0.07 [95% CI: 0.05–0.11], 0.06 [95% CI: 0.05–0.08] and 0.07 [95% CI: 0.04–0.12] for TF SAPIEN™, TA SAPIEN and CoreValve respectively (Fig. 6). (*P* = 0.9). High heterogeneity was found among registries for TF SAPIEN (I^2^ = 84.7%) and CoreValve (I^2^ = 94.4%) but not for TA SAPIEN (I^2^ = 29.1%). However, there was a significant publication bias (*P* = 0.002) and trim and fill method suggested an overall prevalence of aortic paravalvular leak of 0.10 [95% CI: 0.0767–0.131].

### 1 year mortality

**Fig. 7:**
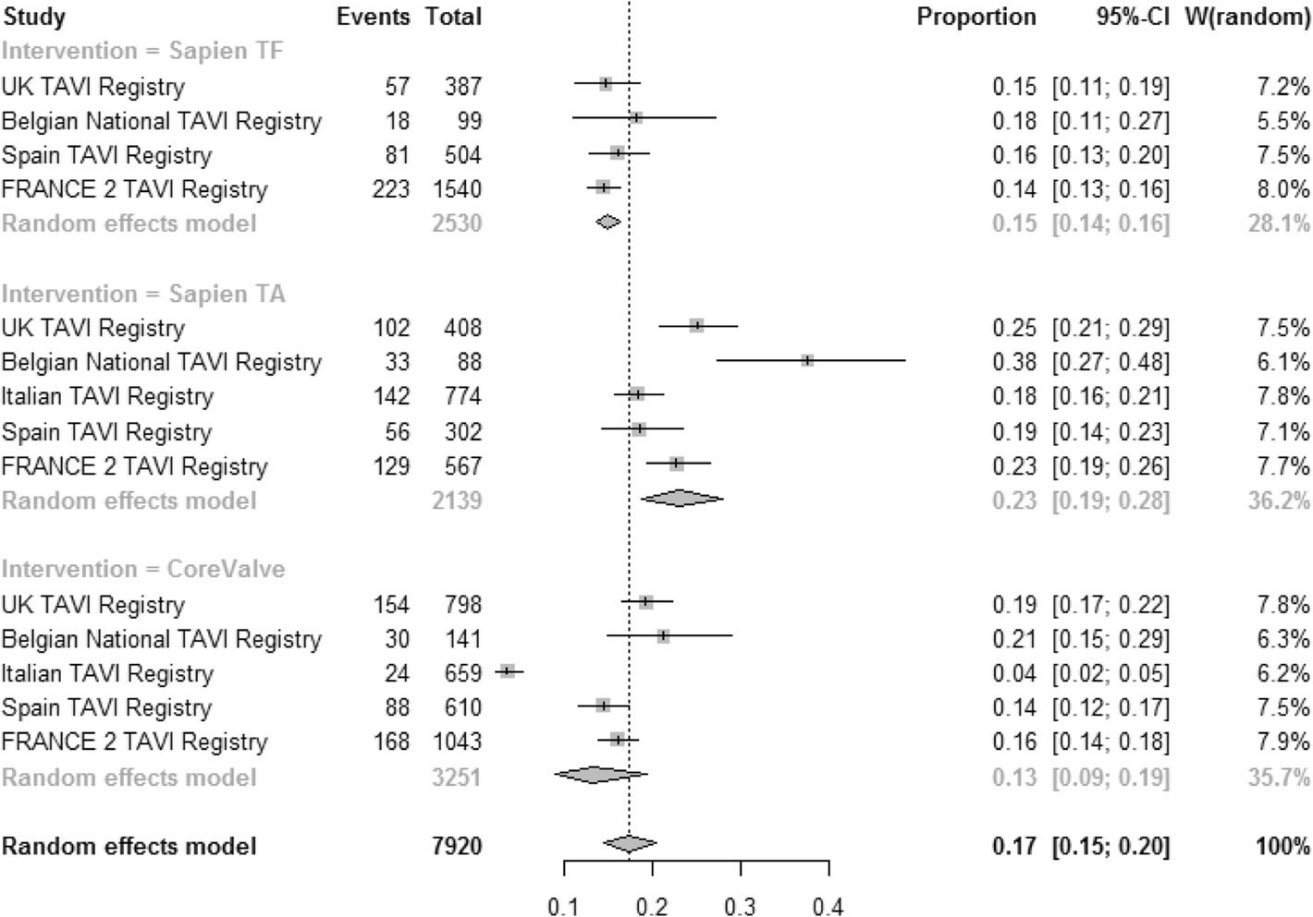
Meta-analysis for proportions of 1-year mortality

Pooled estimate for incidence of 1 year mortality (Fig. 7) was 0.15 [95% CI: 0.14–0.16], 0.23 [95% CI: 0.19–0.28] and 0.17 [95% CI: 0.15–0.19] for TF SAPIEN™, TA SAPIEN and CoreValve respectively (test for subgroup difference *P* = 0.0008). High heterogeneity was found among registries for TA SAPIEN (I^2^ = 81.9%) and CoreValve (I^2^ = 59.3%) but not for TF SAPIEN (I^2^ = 0%). At multivariate meta-regression, mean Logistic EuroSCORE was moderately associated with all-cause mortality at one year (b coefficient 0.07; *P* = 0.06, Fig. 3 right). Different TAVI strategies did not impact on the 1-year mortality (TF SAPIEN versus CoreValve: b coefficient −0.1428; *P* = 0.07) and TA SAPIEN versus CoreValve: b coefficient 0.1515; *P* = 0.3). Moderators included in the multivariate model accounted for 100% amount of heterogeneity and no significant residual heterogeneity was found (*P* = 0.48). No publication bias was detected (*P* = 0.25).

## Discussion

This meta-analysis of all published European TAVI-registries provides an insight to the real world practice of TAVI in the western world. The results support the superiority of the transarterial approach, regardless of the type of implantation device used. CoreValve was associated with > 2 fold increased risk for pacemaker implantation. There was a marked heterogeneity among European countries for all short-term outcomes investigated. Logistic EuroSCORE, which is widely adopted in Europe to select patients at high-risk for surgical AVR, failed to predict 30-day mortality but it was moderately associated with 1-year mortality.

The present meta-analysis found that the overall European TAVI clinical practice has a 30-day mortality of 8% which is higher than the mortality reported by the randomized trials (PARTNER trial A: 3.4% for TF-TAVI, PARTNER trial B: 5% for TF-TAVI and STACCATO trial [16]: 5.8% in TA-TAVI). The inclusion of unselected patients with higher risk profile (mainly severe peripheral disease) in the TA SAPIEN group might partially explain the inferior outcomes and the higher 30-day mortality (12%) for this group.

Particularly with regards to the German registry, there is a substantial difference in mortality across all TAVI strategies and in particular in relation to the TA SAPIEN group, which is difficult to explain in isolation. This may be due to the fact that the German registry is reporting the initial period of introduction of the technique which may not be a true representation of the current status. Isolated published series have independently reported lower mortality for TA-TAVI [17, 18].

It was also evident that, the more concentrated the program was to a limited number of centres, the lower the reported 30-day mortality was for the corresponded country, suggesting that TAVI should be offered by a restricted and highly specialised centres.

Logistic EuroSCORE was not associated with 30-day mortality in the present analysis. It is widely recognized that the logistic EuroSCORE is not an ideal tool for measuring the pre-procedural risk of TAVI as the predicted mortality is grossly overestimated [19]. The present analysis found that early mortality after TAVI varied significantly across European countries, regardless of the type of strategy used. These discrepancies might be partially explained by national differences in patient selection, not accounted for by the logistic EuroSCORE but also by other factors like team integration and learning curve, use of general versus local anaesthesia and level of postoperative care. The presence of heterogeneity in early mortality across European countries highlights the urgent need for standardization of patient selection criteria for TAVI.

The incidence of stroke after TAVI in PARTENR A & B and the STACCATO trials was 5.5, 6.7 and 8.8% respectively. Our meta-analysis has revealed the European incidence of stroke after TAVI is 3.0%, regardless of the TAVI strategy used. This result is encouraging considering the risk of peri-operative stroke following surgical AVR in elderly patients, which is ranging from 3 to 7% [20].

This analysis has also confirmed that CoreValve is associated with an increased rate of pacemaker implantation when compared to the SAPIEN valve (8%). However, the rate of pacemaker implantation in patients receiving SAPIEN valve was higher than those reported in the PARTNER A (3.8%) and B (3.4%) trials. This could be a reflection of the increased anatomic complexity for TAVI in the real-world practice.

This meta-analysis has reported an overall 7% incidence of moderate paravalvular leak (≥2) is better than that the one reported in PARTNER A (12.2%) and B (11.8%) trials. The standardisation of techniques and the increasing familiarity with the procedures with better understanding of deployment strategies may have influence the reduction in the incidence of moderate paravalvular leak, which is the most important complication that negatively influences the long term outcome after TAVI [21]. However, there is an absence of standardized in definition and quantification of paravalvular leak after TAVI (i.e. angiography versus echocardiography, qualitative or semi-quantitative) which needs to be addressed.

Finally, the logistic EuroSCORE was the only moderator marginally associated with 1-year mortality, suggesting that mid-term outcome after TAVI is more “patient-related” rather than “procedure-related”.

This manuscript has a number of limitations; there is a marked heterogeneity in the reported data, which reflect differences in practice, data collection and data analysis. Patient level data were not disclosed and as such, any attempt to analyse further the observed differences is not possible. We understand and acknowledge that meta-analysis of data from different registries remains inferior to RCT. Finally; there is an inherited lack of surgical comparator that makes it difficult for the reader to formulate clear opinion on the subject.

## Conclusions

In conclusion, this meta-analysis of European TAVI registries is reporting higher 30-day mortality than that reported by the published randomized trials, in particular after transapical TAVIs. This result may reflect the inclusion of patients with a higher risk profile. The evidence suggests that transarterial TAVI should be the preferred valve implantation strategy, whenever possible. The significant heterogeneity in early mortality across European countries suggests that there in an urgent need for standardization of patient selection process and procedural aspects in order to optimise outcomes and guarantee high standards of care, across European countries. Despite the fact that in Europe patients are currently selected for TAVI on the basis of their surgical risk measured by the logistic EuroSCORE (≥20%), such a risk stratification system is ineffective in predicting early mortality after TAVI. European registries of TAVI and surgical AVR (with clear differentiation amongst conventional, minimally invasive and suture-less minimally invasive surgical AVR) may be used to generate a well-powered TAVI-weighted risk score.

## Abbreviations

TA: transapical
TAVI: transcatheter aortic valve implantation
TF: transfemoral
